# Cross talk between hedgehog and epithelial–mesenchymal transition pathways in gastric pit cells and in diffuse-type gastric cancers

**DOI:** 10.1038/sj.bjc.6604846

**Published:** 2008-12-23

**Authors:** H Ohta, K Aoyagi, M Fukaya, I Danjoh, A Ohta, N Isohata, N Saeki, H Taniguchi, H Sakamoto, T Shimoda, T Tani, T Yoshida, H Sasaki

**Affiliations:** 1 Genetics Division, National Cancer Center Research Institute, 1-1, Tsukiji 5-chome, Chuo-ku, Tokyo 104-0045, Japan; 2 Pathology Division, National Cancer Center Research Institute, 1-1, Tsukiji 5-chome, Chuo-ku, Tokyo 104-0045, Japan; 3 Clinical Laboratory, National Cancer Center Hospital, 1-1, Tsukiji 5-chome, Chuo-ku, Tokyo 104-0045, Japan; 4 Department of Surgery, Shiga University of Medical Science, Seta Tsukinowa-cho, Otsu-shi, Shiga 520-2192, Japan

**Keywords:** gastric pit cell, diffuse-type gastric cancer, hedgehog, epithelial–mesenchymal transition, cancer-linked hypomethylation

## Abstract

We previously reported hedgehog (Hh) signal activation in the mucus-secreting pit cell of the stomach and in diffuse-type gastric cancer (GC). Epithelial–mesenchymal transition (EMT) is known to be involved in tumour malignancy. However, little is known about whether and how both signallings cooperatively act in diffuse-type GC. By microarray and reverse transcription–PCR, we investigated the expression of those Hh and EMT signalling molecules in pit cells and in diffuse-type GCs. How both signallings act cooperatively in those cells was also investigated by the treatment of an Hh-signal inhibitor and siRNAs of Hh and EMT transcriptional key regulator genes on a mouse primary culture and on human GC cell lines. Pit cells and diffuse-type GCs co-expressed many Hh and EMT signalling genes. Mesenchymal-related genes (*WNT5A*, *CDH2*, *PDGFRB*, *EDNRA*, *ROBO1*, *ROR2*, and *MEF2C*) were found to be activated by an EMT regulator, SIP1/ZFHX1B/ZEB2, which was a target of a primary transcriptional regulator GLI1 in Hh signal. Furthermore, we identified two cancer-specific Hh targets, *ELK1* and *MSX2*, which have an essential role in GC cell growth. These findings suggest that the gastric pit cell exhibits mesenchymal-like gene expression, and that diffuse-type GC maintains expression through the Hh–EMT pathway. Our proposed extensive Hh–EMT signal pathway has the potential to an understanding of diffuse-type GC and to the development of new drugs.

Gastric cancer (GC) is one of the most frequent fatal malignancies in the world (Maxwell Parkin, 2001), and will be unresectable for more than two-thirds of its sufferers. Even patients with an operable tumour have a high rate of both local and distant recurrence with a 5-year survival rate of less than 30%; furthermore, the response rate to chemotherapy in unresectable and recurrent cases is at most 40% (Wohrer *et al*, 2004; Lordick and Siewert, 2005). Gastric cancers are histologically classified into two major types: intestinal-type (clustered and well-differentiated, and glandular-like types) and diffuse-type (infiltrating, poorly-differentiated, and scattered types) (Lauren, 1965; Tatematsu *et al*, 1990; Ming, 1998; Yuasa, 2003). Intestinal-type GC develops through some sequential stages including *Helicobacter pylori (H. pylori)*-associated gastritis, intestinal metaplasia (IM), and dysplasia. This type predominates in high-risk geographic areas, such as east Asia, showing a correlation with the prevalence in the region of *H. pylori* infection among the elderly. Diffuse-type GC, however, is more uniformly distributed geographically, is apparently unrelated to *H. pylori* prevalence and typically develops from *H. pylori*-free, morphologically normal gastric mucosa without atrophic gastritis, or IM. A *de novo* diffuse-type GC is believed to develop from stem cells or precursors for gastric epithelial cells in the background of relatively normal gastric mucosa (Hohenberger and Gretschel, 2003; Schier and Wright, 2005). Unlike the decreasing incidence of the intestinal-type GC, the prevalence of the diffuse type is reportedly increasing worldwide (Crew and Neugut, 2006). Therefore, molecular characterisation of diffuse-type GC, especially on infiltrating and scattered type of growth or identification of cancer stem cell is important for the development of new drugs for this type of cancer. The infiltrating and scattered type of growth in diffuse-type GC has been reported to be mediated by loss of E-cadherin (CDH1) function through somatic mutation, promoter methylation, and cancer-associated downregulation (Kountouras *et al*, 2005). Recently, CDH1 has been reported to be downregulated by an epithelial–mesenchymal transition (EMT) regulator, TWIST, which plays an essential role in breast cancer metastasis, especially of the diffuse type (Yang *et al*, 2004). However, a more detailed signal network needs to be revealed to understand diffuse-type GC growth and progression.

Recently, we reported that hedgehog (Hh) signal activation selectively occurs in diffuse-type GC and that the Hh signal block inhibits growth of GC cells with Hh activation (Fukaya *et al*, 2006). In mammals, Hh ligands are composed of three members: Sonic Hh (SHH), Indian Hh (IHH), and Desert Hh (DHH). Activation of Hh signalling is initiated through binding of any of the above three ligands to a 12-transmembrane protein receptor, PTCH, which acts as a negative regulator of a 7-transmembrane protein, SMO. Binding between Hh and PTCH results in de-repression of SMO, thereby activating a cascade that leads to the translocation of the active form of the transcription factor GLI to the nucleus. Nuclear GLI activates expression of a variety of target genes such as *BMP4*, *FOXA2*, *ISL1*, and *FOXM1* (Krishnan *et al*, 1997; Van den Brink *et al*, 2001; Teh *et al*, 2002). Recently, Ihog (interference Hh) and Boi, which de-repress SMO through interaction with Hh ligands, have been identified in *Drosophila* (Yao *et al*, 2006). Their mouse/human homologues Boc/BOC and Cdo/CDO have the same functions (Tenzen *et al*, 2006; Yao *et al*, 2006; Zhang *et al*, 2006).

Here we report that an EMT regulator SIP1 is a target of the Hh signal in gastric pit cells and in diffuse-type GC, and that SIP1 regulates mesenchymal-related genes (*WNT5A, CDH2, PDGFRB, EDNRA, ROBO1, ROR2,* and *MEF2C*), which express preferentially in both pit cell and diffuse-type GC. Furthermore, we identified two cancer-specific Hh targets, *ELK1* and *MSX2*, which have an essential role in tumour cell growth.

## Materials and methods

### Tissue samples

Gastric cancer and non-cancerous tissues were provided by the National Cancer Center Hospital after obtaining informed consent from each patient and approval by the Center's Ethics Committee. All cancer specimens were reviewed and classified histopathologically according to the Japanese Classification of Gastric Cancer. Tissue specimens were immediately frozen with liquid nitrogen after surgical extraction, and stored at −80°C until use.

### Microarray analysis

Total RNA was isolated by suspending the cells in an ISOGEN lysis buffer (Nippon Gene, Toyama, Japan), followed by precipitation with isopropanol. We used Human Expression Array U95A version 2 (Affymetrix, Santa Clara, CA, USA) for analysis of mRNA expression levels corresponding to 12 600 transcripts. The procedures were conducted according to the supplier's protocols. The expression value (average difference; AD) of each gene was calculated using GeneChip Analysis Suite version 4.0 software (Affymetrix). The mean of AD values in each experiment was 1000 to reliably compare variable multiple arrays.

### Laser microdissection (LMD), RNA extraction, RT–PCR, and quantitative real-time PCR

The cryostat sections (8 *μ*m) of frozen tissues were microdissected with a Pixcell II LCM system (Arcturus Engineering, Mountain View, CA, USA). Total RNA was isolated by suspending the cells in an ISOGEN lysis buffer (Nippon Gene), followed by precipitation with isopropanol. The mRNA was amplified by an efficient method of high-fidelity mRNA amplification, called TALPAT (T7 RNA polymerase promoter-attached, adaptor ligation-mediated, and PCR amplification followed by *in vitro* T7-transcription) (Aoyagi *et al*, 2003; Kobayashi *et al*, 2004; Nakamura *et al*, 2006). As described in our previous report (Fukaya *et al*, 2006), semi-quantitative reverse transcription (RT)–PCR and quantitative real-time PCR were carried out using primer sets (Supplementary Table 1). For semi-quantitative RT–PCR, we showed data within linear range by performing 25–35 cycles of PCR. For quantitative real-time PCR, the expression level of each mRNA was normalised with that of *GAPDH* mRNA.

### Immunohistochemistry

Specimens fixed in formalin and embedded in paraffin were cut into 4 *μ*m sections, subsequently dewaxed, and dehydrated. Endogenous peroxidase activity was blocked with 3% H_2_O_2_ in methanol for 30 min and endogenous biotin with a blocking kit (Vector Laboratories, Burlingame, CA, USA). Antigen retrieval was performed by autoclave for 10 min at 121°C in 10 mM citrate buffer, pH 6.0. Sections were blocked for DAKO protein block (DAKO, Carpinteria, CA, USA), and incubated overnight at 4°C with diluted rabbit polyclonal antibody directed against human GLI1 (sc-20687, 1 : 100; Santa Cruz Biochemistry, Santa Cruz, CA, USA), GLI2 (ab7181, 1 : 200; Abcam Ltd, Cambridge, MA, USA), PDGFRB (sc-339, 1 : 200; Santa Cruz Biochemistry), EDNRA (E3651, 1 : 100; SIGMA, St Louis, MO, USA), and mouse monoclonal antibody directed against SMTN (MAB3242, 1 : 200; Chemicon International, Tamecula, CA, USA). The next day, after washing the sections with PBS containing 0.1% Tween-20, biotinylated secondary antibodies were added for 30 min at room temperature. Detection was carried out with the Vectastain ABC Elite Kit (Vector Laboratories). After extensive rinsing and incubation with an avidin-biotin immunoperoxidase complex, staining was visualised with the DAB system (Nichirei, Tokyo, Japan), and the sections were counter-stained with Mayer's hematoxylin.

### Cell lines and siRNA transfection

Seven diffuse-type GC-derived cell lines, HSC39, HSC43, HSC44, HSC58, HSC59, HSC60, and KATOIII, and four intestinal-type GC-derived cell lines, MKN7, MKN28, MKN74, and HSC57 were maintained in RPMI1640 or Dulbecco's modified Eagle's medium supplemented with 10% fetal calf serum, 0.15% sodium bicarbonate, 2 mM L-glutamine, and penicillin-streptomycin. An Hh-pathway-specific antagonist cyclopamine or tomatidine, an inactive but structurally related compound (Toronto Research Chemicals, North York, Ontario, Canada) dissolved in 100% ethanol was added to HSC60 at 30 *μ*M. After 6, 12, and 24 h, cells were collected and total RNA was isolated, respectively. Five siRNA fragments were designed for suppressing *GLI1, GLI2, SIP1, ELK1,* and *MSX2* expression respectively, and the most effective one was selected by quantitative real-time RT–PCR analysis. The five siRNAs were *GLI1* siRNA (SI00074802, QIAGEN, Valencia, CA, USA), *GLI2* siRNA (SI02634842, QIAGEN), *SIP1* siRNA (108633, Ambion, Austin, TX, USA), *ELK1* siRNA (SI00300146, QIAGEN), and *MSX2* siRNA (SI00038031, QIAGEN). These siRNAs were introduced to HSC60 using Dharma FECT1™ (Dharmacon, La Fayette, CO, USA), following the procedure recommended by the manufacturer. The RT–PCR analysis was carried out at 24 h after siRNA transfection.

### Primary culture of mouse gastric epithelial cells

On postnatal day 4, the stomach of 57BL/6J mice was minced, then suspended and sterilised with 10% antibiotic–antimycotic drugs (Invitrogen, Carlsbad, CA, USA), and digested by incubation for 12 min at 37°C in 0.05%. collagenase 1 (Nitta Gelatine, Osaka, Japan), and then manually dissociated with scissors, and incubated for 5 min at 37°C with a 1 : 1 ratio of 0.5% Trypsin-EDTA (Invitrogen) and Dispase 10000PU (Godo Shusei, Tokyo, Japan) in PBS (−). After being filtered with a 100 *μ*m cell strainer (Falcon, Franklin Lakes, NJ, USA), the digested tissue fragments were centrifuged for 5 min at 1200 r.p.m., resuspended in Defined Keratinocyte-SFM (Invitrogen) with 1 ml of supplement and antibiotic–antimycotic, plated in type 1 collagen-coated 35 mm culture dishes (IWAKI, Tokyo, Japan) and incubated at 37°C in a humidified atmosphere flushed with 5% CO_2_ in the air. Twenty-four hours after plating, non-adhesive cells were discarded. At 1 day after culture, 10 *μ*M of cyclopamine or tomatidine was added.

### Matrigel invasion assay

Invasion of the GC HSC60 cells *in vitro* was measured by BD BioCoat™ Matrigel™ Invasion Chamber (6-well) (Becton Dickinson Biosciences, Bedford, MA, USA), according to the manufacturer's protocol. After *SIP1* siRNA transfection, the cells were trypsinised and 2 ml of cell suspension (2 × 10^5^ cells ml^−1^) was added in triplicate wells. For a cell growth assay, we counted the number of cells at 24 h after *SIP1* siRNA transfection. For an invasion assay, the cells that passed through the filter into the lower wells were fixed and stained with 100% methanol and 1% Toluidine blue, respectively. The number of invading cells was counted through a microscope at 24 h after *SIP1* siRNA transfection.

## Results

### Genome-wide mRNA expression profiling of primary intestinal-type and diffuse-type GCs

To identify the specific genes for each type of GC, we selected 18 intestinal-type GCs and 12 diffuse-type GCs showing typical characteristics on the form of cell growth (clustered or scattered) and the degree of differentiation (well/moderate or poor), and performed microarray analysis for obtaining genome-wide mRNA expression profiles. First, we conducted unsupervised clustering analyses using some gene sets, which were differentially expressed depending samples. Most of the 12 diffuse-type GCs were able to separate from the 18 intestinal-type GCs (data not shown), suggesting the presence of a distinct difference on expression profiles between the two types. Next, to compile a gene list for identifying diffuse-type GC-specific signal pathways, we selected genes by comparing the expression levels of the two types. A gene was selected by Wilcoxon *U*-test (*P*<0.05) from genes with more than a two-fold change on average. By this procedure, a total of 892 genes (704 genes specific to diffuse-type and 188 genes specific to intestinal-type) were identified. The result of a two-dimensional hierarchical clustering analysis of the 892 selected genes is shown in Supplementary Figure 1. In the 188 intestinal-type-specific genes, *CCNDE*, *ERBB2*, and *GRB7*, which have been reported to be amplified and overexpressed in intestinal-type GC (Yasui *et al*, 2001), were included. In the 704 diffuse-type-specific genes, three GLI1-target genes *FOXM1*, *ISL1*, and *FOXF2* were included in accordance with our previous report (Fukaya *et al*, 2006). More interestingly, *CDH1* encoding an epithelial cell marker E-cadherin, which has been reported to be downregulated by an EMT regulator, TWIST, in breast cancer (Yang *et al*, 2004), was found only in intestinal-type GC, suggesting that diffuse-type GC may show EMT. In accordance with this suggestion, nine other mesenchymal-related genes (three muscle-related genes, *SMTN, LMOD*, and *MEF2C*; one blood-related gene, *PDGFRB*; one neuronal endocrine-related gene, *EDNRA*; two endothelium-related genes, *SLIT2* and *ROBO1*; a target gene of an EMT regulator SIP1, *WNT5A* and its receptor, *ROR2*), were also found in the diffuse-type-specific gene list, although expression of EMT regulator genes (*TWIST1*, *TWIST2*, and *SNAI2*) other than *SIP1* was never detected in the microarray platform used.

### Normal gastric pit cells and diffuse-type GCs possess mesenchymal-like gene expression

In the gastric corpus, the epithelium consists of three tubular units from surface to base: a pit region containing mucus-secreting pit cells, an isthmus/neck region containing stem cells, and a gland region containing chief and parietal cells (Karam and Leblond, 1995). Intestinal-type GC is thought to develop from IM by transdifferentiation, whereas diffuse-type GC is derived from stem cell or the pit cell precursor (Ming, 1998; Yuasa, 2003). Therefore, genes specifically expressed in the diffuse-type may be expressed preferentially in the pit region among the three regions of the gastric mucosa. Accordingly, we prepared RNA from each region by LMD for RT–PCR as described in Materials and Methods. We conducted semi-quantitative RT–PCR analysis of Hh signalling- and EMT-related molecules in the three regions of the gastric mucosa, IM, and two types of GC tissues. For semi-quantitative RT–PCR, we performed 25–35 cycles of PCR, and showed data within linear range.

**Figure 1:**
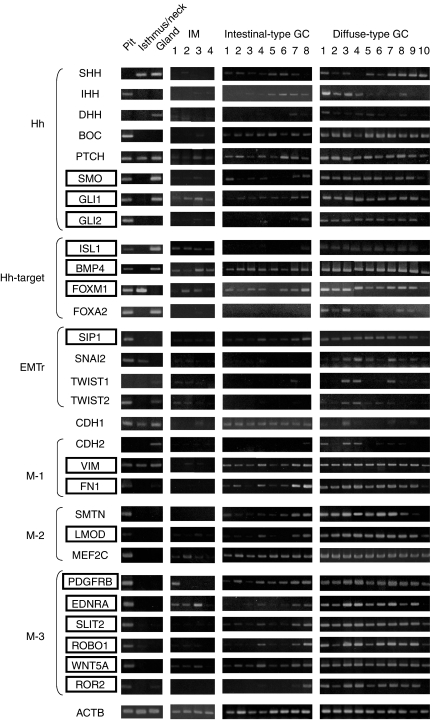
Semi-quantitative RT–PCR analyses of Hh signalling molecules, EMT regulators, and mesenchymal-related genes in three regions (pit, isthmus/neck, and gland) of normal gastric mucosa, intestinal metaplasias (IMs), and two types of gastric cancers. Hh signalling molecules are expressed preferentially in diffuse-type gastric cancers compared with IMs and intestinal-type gastric cancers, as reported previously (Fukaya *et al*, 2006). Three EMT regulators (*SIP1, SNAI2,* and *TWIST2*) and 10 mesenchymal-related genes (*FN1, SMTN, LMOD, MEF2C, PDGFRB, EDNRA, SLIT2, ROBO1, WNT5A,* and *ROR2*) are preferentially expressed in the pit region. These EMT regulators and mesenchymal-related genes are expressed highly and preferentially in diffuse-type gastric cancers compared with the IMs and intestinal-type gastric cancers. EMTr, EMT regulator; M-1, three typical mesenchymal genes; M-2, three muscle-related mesenchymal genes, and M-3, six other mesenchymal-related genes. GC, gastric cancer.

Shown in Figure 1 are the results of semi-quantitative RT–PCR on eight Hh signalling genes (*SHH, IHH, DHH, BOC, PTCH, SMO, GLI1,* and *GLI2*), four GLI1 targets (*ISL1, BMP4, FOXM1,* and *FOXA2*), four EMT regulator genes (*SIP1, SNAI2, TWIST1,* and *TWIST2*), an epithelial cell marker (*CDH1*), three mesenchymal markers (*CDH2, VIM,* and *FN1*, M-1 in Figure 1), and nine other mesenchymal-related genes (*SMTN*, *LMOD*, *MEF2C*, *PDGFRB*, *EDNRA*, *SLIT2*, *ROBO1*, *WNT5A*, and *ROR2*, M-2 and M-3 in Figure 1), in the RNA samples from the three regions of gastric mucosa, four IMs, eight intestinal-type GCs and 10 diffuse-type GCs.

In our previous report (Fukaya *et al*, 2006), preferential expression of most of the Hh signalling molecules (*SHH, IHH, DHH, PTCH, SMO, GLI1, GLI2*, *ISL1, BMP4, FOXM1,* and *FOXA2*) in diffuse-type GCs compared with IMs and intestinal-type GCs is confirmed by quantitative real-time RT–PCR in the same sample sets used in this paper. A new positive mediator of Hh signalling *BOC* expressed highly in diffuse-type GCs, while another positive mediator *CDO* expressed ubiquitously in the three regions of the gastric mucosa, IM, and two types of GC tissues (data not shown). The epithelial cell marker *CDH1* was downregulated in the diffuse-type compared with the intestinal-type.

In normal tissue, among the four EMT regulators, *SIP1, SNAI2,* and *TWIST2* were preferentially expressed in the pit region, whereas *TWIST1* was expressed only in the gland region. In correspondence with *SIP1, SNAI2*, and *TWIST2* expressions in the pit region, *FN1, SMTN, LMOD, MEF2C, PDGFRB, EDNRA, SLIT2, ROBO1, WNT5A,* and *ROR2*, preferentially expressed in the pit region, whereas *CDH2* expressed in the gland region concordant with *TWIST1* expression. In abnormal and malignant tissue, EMT regulators and mesenchymal-related genes were downregulated frequently in both the IMs and intestinal-type GCs, while those expressions were maintained in the diffuse-type GCs.

**Figure 2:**
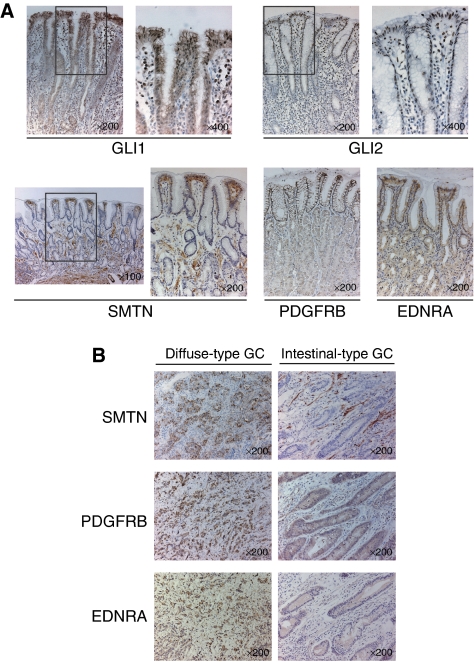
Immunohistochemistry of Hh downstream targets (GLI1 and GLI2) and mesenchymal-related genes (SMTN, PDGFRB, and EDNRA) in normal gastric mucosas and in gastric cancer tissues. (**A**) Both GLI1 and GLI2 are localised in the nuclei of the pit cells, and SMTN, PDGFRB, and EDNRA are stained preferentially in the cytoplasm or cell membrane of the pit cells. (**B**) SMTN, PDGFRB, and EDNRA are stained strongly in diffuse-type gastric cancer cells compared with intestinal-type gastric cancer cells.

The results of RT–PCR analysis were confirmed by immunohistochemical analyses with GLI1, GLI2, SMTN, PDGFRB, and EDNRA antibodies. Both GLI1 and GLI2 were localised in the nuclei of the pit cells, and SMTN, PDGFRB, and EDNRA were stained preferentially in the cytoplasm or membranes of the pit cells (Figure 2A). These three genes were detected clearly in diffuse-type GC cells, but scarcely detected in intestinal-type GC cells (Figure 2B). These results suggest that, despite a differentiated epithelial cell, the gastric pit cell exhibits the mesenchymal phenotype, and that diffuse-type GC also maintains it. This suggests the presence of cross talk between the Hh and EMT signal pathways in both the pit cell and diffuse-type GC.

### Cross talk between Hh and EMT pathways in gastric pit cells and in diffuse-type GCs

As shown in Figure 1, in GC tissues, expression patterns of Hh- and EMT-related genes including a Hh-primary transcriptional target *GLI1* and an EMT regulator *SIP1* (indicated by a box) were most likely to be similar among the samples examined (only the no. 10 case in 10 diffuse-type GCs shows weak expression of these genes, whereas nos. 1, 2, 3, 5, and 6 cases in eight intestinal-type GCs showed weak). This observation suggested the presence of cross talk between Hh and EMT pathways.

**Figure 3:**
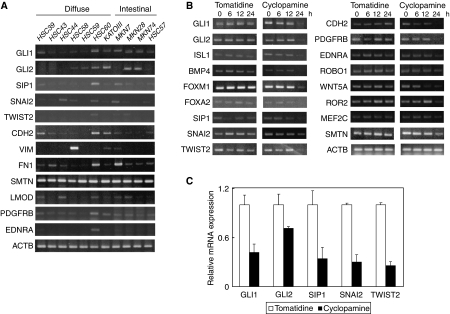
Effect of treatment of Hh-signal-specific inhibitor, cyclopamine on gastric cancer cells. (**A**) RT–PCR analyses of Hh- and EMT-related genes in 11 gastric cancer cell lines. Only HSC60 cells most express Hh signalling, EMT regulator, and mesenchymal genes. (**B**) Cyclopamine treatment reduces mRNA of not only six Hh-downstream genes (*GLI1, GLI2, ISL1, BMP4, FOXM1,* and *FOXA2*) but also three pit cell-expressing EMT regulators (*SIP1, SNAI2,* and *TWIST2*) and eight mesenchymal-related genes (*CDH2, PDGFRB, EDNRA, ROBO1, WNT5A, ROR2*, *MEF2C*, and *SMTN*). (**C**) Quantitative real-time RT–PCR analyses of Hh-downstream genes (*GLI1* and *GLI2*) and EMT regulators (*SIP1, SNAI2* and *TWIST2*) show the same results of the above semi-quantitative RT–PCR. Results were calculated as mean+s.d. values from triplicate measurements of three separate experiments.

To examine whether the EMT regulator gene is a downstream target of GLIs in Hh signalling, we first selected the GC cell line, which most-closely mimics the normal pit cell and diffuse-type GC phenotype in mRNA expression of Hh- and EMT-related genes among 11 GC-derived cell lines. As shown in Figure 3A, the diffuse-type GC cell lines except a HSC60 cell line (HSC39, HSC 43, HSC44, HSC58, HSC59, and KATOIII) appeared to have downregulated expression of a number of the Hh- and EMT-related genes. A tumour tissue provides various microenvironments (low nutrition, hypoxia, and so on) for tumour cell growth. In a tumour tissue, diffuse-type GC cells show scattered type growth by interaction with myofibroblasts. In a cell culture, three cell lines (HSC39, HSC58, and KATOIII) grow as spheroids. However, this characteristic could not explain the downregulation in the cell lines. Accordingly, the microenvironment and/or the interaction may be required for the activation of the Hh and EMT pathways. As shown in Supplementary Figure 2, both *GLI1* and *SIP1* were never upregulated in a culture with a low serum level. Recently, hypoxia-inducing factor-1 (HIF-1) has been reported to activate or stabilise EMT regulators including TWIST and SNAIL (Gort *et al*, 2008; Sahlgren *et al*, 2008; Yang *et al*, 2008), but no report for such activation in GLIs has been found. Therefore, to date, the difference of EMT-related gene expression between cultured cells and primary tumours can be postulated partly by hypoxia. Although it was still unknown why the HSC60 cell line maintains the predisposition of both the pit cell and diffuse-type GC in expression of Hh signal- and EMT-related genes (Figure 3A), to verify the above-mentioned hypothesis, we examined the effects of treatment of an Hh signal pathway-specific inhibitor, cyclopamine (Cooper *et al*, 1998; Chen *et al*, 2002) in this cell line. The mRNA level of not only six Hh-downstream genes (*GLI1*, *GLI2*, *ISL1*, *BMP4*, *FOXM1,* and *FOXA2*) but also three pit cell-expressing EMT regulators (*SIP1, SNAI2*, and *TWIST2*) and their candidate downstream genes (*CDH2, PDGFRB, EDNRA, ROBO1, WNT5A, ROR2, MEF2C,* and *SMTN*) significantly decreased at 24 h after cyclopamine treatment compared with tomatidine, an inactive but structurally related compound (Figure 3B). The results of semi-quantitative RT–PCR of five key transcription factors (*GLI1*, *GLI2*, *SIP1*, *SNAI2,* and *TWIST2*) were confirmed by performing quantitative real-time PCR (Figure 3C).

**Figure 4:**
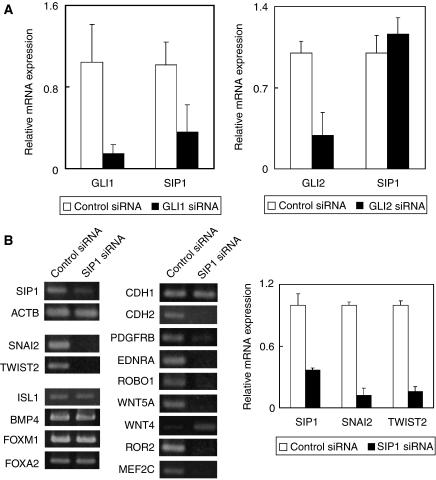
The EMT regulator gene SIP1 is a mediator for mesenchymal-like gene expression through Hh signalling in diffuse-type gastric cancer cells. (**A**) Quantitative real-time RT–PCR analyses of *SIP1* mRNA in *GLI1* or *GLI2* siRNA-treated HSC60 cells. *GLI1* siRNA reduces *SIP1* mRNA, whereas *GLI2* siRNA does not reduce *SIP1* mRNA. Results were calculated as mean+s.d. values from triplicate measurements of three separate experiments. (**B**) Conventional and quantitative real-time RT–PCR analyses of EMT regulators, Hh target genes, and mesenchymal-related genes in *SIP1* siRNA-transfected HSC60 cells. In accordance with a decrease of *SIP1* mRNA, other EMT regulators (*SNAI2* and *TWIST2*), and various mesenchymal-related genes (*CDH2, PDGFRB, EDNRA, ROBO1, WNT5A, ROR2,* and *MEF2C*) are downregulated. On the other hand, mRNA levels of Hh target genes (*ISL1, BMP4, FOXM1,* and *FOXA2*) and *CDH1*, an epithelial cell marker, are never affected, and *WNT4*, which is the other epithelial cell marker and involved in mesenchymal–epithelial transition (MET) (Stark *et al*, 1994), is clearly upregulated by *SIP1* siRNA treatment. Results of quantitative real-time RT–PCR were calculated as mean+s.d. values from triplicate measurements of three separate experiments.

Consecutively, *GLI1* and *GLI2* siRNA transfection were performed to investigate whether SIP1 is a downstream target of GLI. *GLI1* siRNA transfection reduced the mRNA level of *SIP1*, whereas *GLI2* siRNA transfection never affected it (Figure 4A). These results suggest that *SIP1* is a downstream gene of GLI1 but not of GLI2, although a decrease of SIP1 protein could not be confirmed due to the low amount of SIP1 in HSC60 and the low specificity of anti-SIP1antibodies obtained commercially and by immunisation of our prepared-peptide sequences (data not shown). Next, we conducted *SIP1* siRNA transfection to examine the downstream pathway of SIP1 (Figure 4B). *SIP1* siRNA treatment reduced mRNAs of *SIP1*, two other EMT regulators (*SNAI2*, and *TWIST2*) and seven other genes (*CDH2, PDGFRB, EDNRA, ROBO1, WNT5A, ROR2,* and *MEF2C*). Although an epithelial cell marker *CDH1* was never affected by *SIP1* siRNA treatment, *WNT4*, which has been reported to be involved in epithelial transition (Stark *et al*, 1994), was clearly upregulated by the treatment (Figure 4B, left panel).

As expected, the Hh target genes (*ISL1*, *BMP4*, *FOXM1*, and *FOXA2*) were never affected by *SIP1* siRNA treatment (Figure 4B, left panel). The results of conventional RT–PCR in *SIP1, SNAI2,* and *TWIST2* were verified by quantitative real-time PCR (Figure 4B, right panel). Those results of cyclopamine- (Figures 3B and 3C), GLI1 siRNA- (Figure 4A), and *SIP1* siRNA (Figure 4B)-treatment suggested that some mesenchymal phenotypes in diffuse-type GC was exhibited by an EMT regulator SIP1 through Hh signalling.

**Figure 5:**
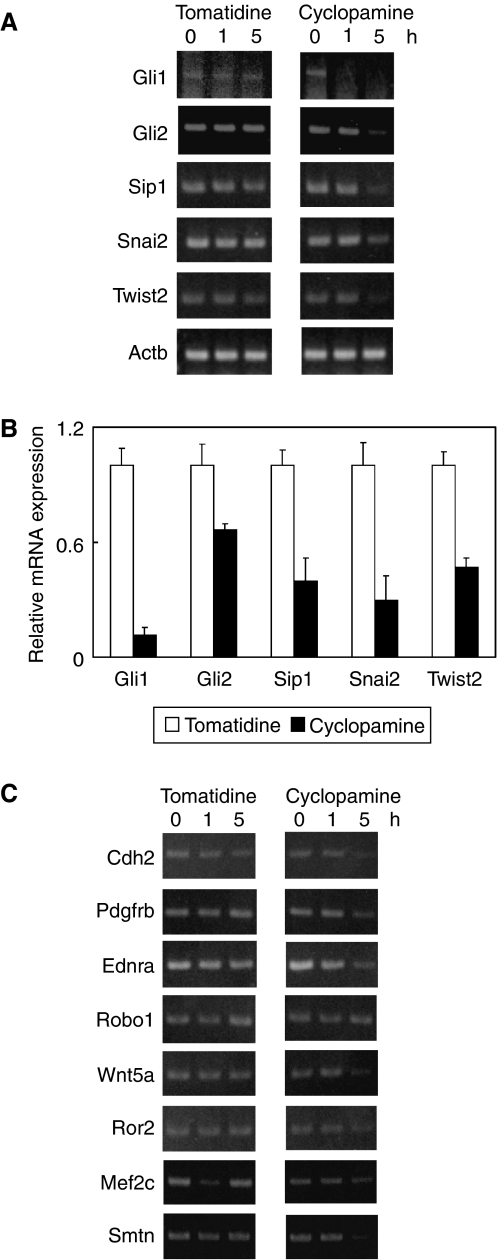
Cyclopamine treatment in mouse primary culture of the gastric epithelial cells. (**A**) In mice as well as in humans, not only Hh-downstream targets (*Gli1* and *Gli2*) but also EMT regulators (*Sip1, Snai2,* and *Twist2*) are downregulated by cyclopamine treatment. (**B**) Quantitative real-time RT–PCR analyses show the same results of the above conventional RT–PCR. Results were calculated as mean+s.d. values from triplicate measurements of three separate experiments. (**C**) Other mesenchymal-related genes (*Cdh2, Pdgfrb, Ednra, Robo1, Wnt5a, Ror2, Mef2c,* and *Smtn*) are also downregulated by cyclopamine treatment. As a control, *Actb* is shown in Figure 5A.

The expression pattern of three ligands, Ihh (pit), Shh (gland), and Dhh (gland) in mouse gastric mucosa is similar to that in human beings, and Hh signal activation is found in both pit cells and parietal cells in murine glandular stomach (Van den Brink *et al*, 2001; Fukaya *et al*, 2006). To this end, to investigate the function of the Hh signal pathway in normal gastric mucosa, we examined the effects of cyclopamine treatment in mouse primary culture of gastric epithelial cells. Cyclopamine treatment reduced mRNAs of not only pit cell expressing-Hh signalling molecules (Gli1 and Gli2) but also EMT regulators (Sip1, Snai2, and Twist2) (Figure 5A). Quantitative real-time PCR analyses showed the same results (Figure 5B). Furthermore, mesenchymal-related genes (Cdh2, Pdgfrb, Ednra, Robo1, Wnt5a, Ror2, Mef2c, and Smtn) were also downregulated by cyclopamine treatment (Figure 5C). These data suggest that mesenchymal-like gene expression of the pit cell in both mice and humans is induced by Hh signal activation.

### Identification of cancer-specific Hh-downstream target genes

**Figure 6:**
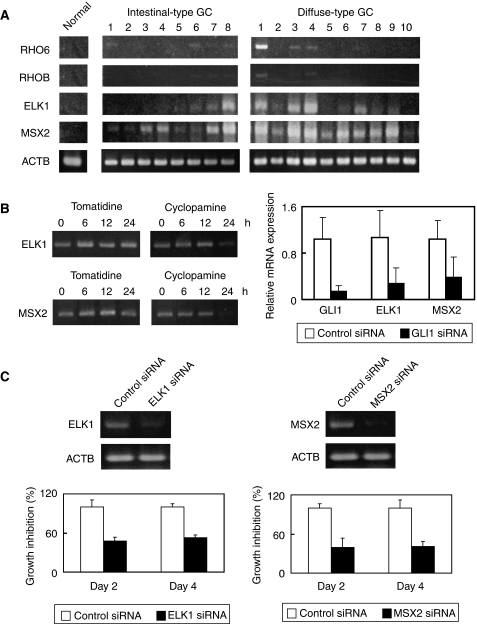
Identification of cancer-specific Hh-target genes. (**A**) RT–PCR analyses of gastric cancer-linked DNA hypomethylated candidate genes (*RHO6, RHOB, ELK1,* and *MSX2*) in normal gastric mucosa and cancer tissues. All four genes are suppressed in normal epithelium, and two genes *ELK1* and *MSX2* of the four genes expressed preferentially in diffuse-type gastric cancer. (**B**) Cyclopamine (left panel) and *GLI1* siRNA (right panel) treatment reduces *ELK1* and *MSX2* mRNAs at 24 h after treatment. Results of real-time RT–PCR were calculated as mean+s.d. values from triplicate measurements of three separate experiments. As a control, *Actb* is shown in Figure 3B. (**C**) Treatment of *ELK1* siRNA and *MSX2* siRNA induces growth inhibition of HSC60 cells (lower panel) in accordance with a decrease of *ELK1* and *MSX2* mRNAs (upper panel). Results of cell growth inhibition assays were calculated as mean+s.d. values from triplicate measurements of three separate experiments. GC, gastric cancer.

An Hh-signal-specific inhibitor, cyclopamine, induces growth inhibition in Hh-activating GC cells; meanwhile in normal gastric epithelium, cyclopamine causes a block of pit cell differentiation followed by epithelial hyperplasia (Fukaya *et al*, 2006). For minimising the side effects in Hh-signal targeted cancer therapy, identification of cancer-specific Hh-downstream genes such as oncogenes is needed. Recently, evidence showing oncogene activation by cancer-linked DNA hypomethylation has accumulated (Feinberg *et al*, 2006). We previously reported 159 genes, including oncogenes, which could be activated by GC-linked DNA hypomethylation (Nishigaki *et al*, 2005). Among the gene list, RT–PCR of four oncogenes (*RHO6, RHOB, ELK1,* and *MSX2*) in surgical specimens was performed. All four genes were suppressed in normal gastric epithelium. Two genes, *ELK1* and *MSX2,* of the four genes were found to express preferentially in diffuse-type GC (Figure 6A). Immunohistochemical analyses confirmed preferential expression of ELK1 and MSX2 proteins in diffuse-type GC cells compared with intestinal-type GC cells. Representative results are shown in Supplementary Figure 3. As shown in Figure 6B, cyclopamine treatment reduced mRNA of *ELK1* and *MSX2*, suggesting both of the genes are regulated by Hh signalling. *GLI1* siRNA treatment reduced mRNA of *ELK1* and *MSX2* (Figure 6B). These results suggest that *ELK1* and *MSX2* may be demethylated in diffuse-type GCs and then activated by GLI1. Next, we investigated whether knockdown of *ELK1* and *MSX2* using siRNA affects cell growth. Treatment of each siRNA of *ELK1* and *MSX2* induced growth inhibition (53 and 41%, respectively) of HSC60 at 4 days after transfection (Figure 6C). Double transfection of these two siRNAs strongly inhibited cell growth (Supplementary Figure 4). These data suggest that blocking of both the ELK1 and MSX2 function could be an effective Hh-targeted cancer therapy.

### SIP1 is involved in GC cell invasion

**Figure 7:**
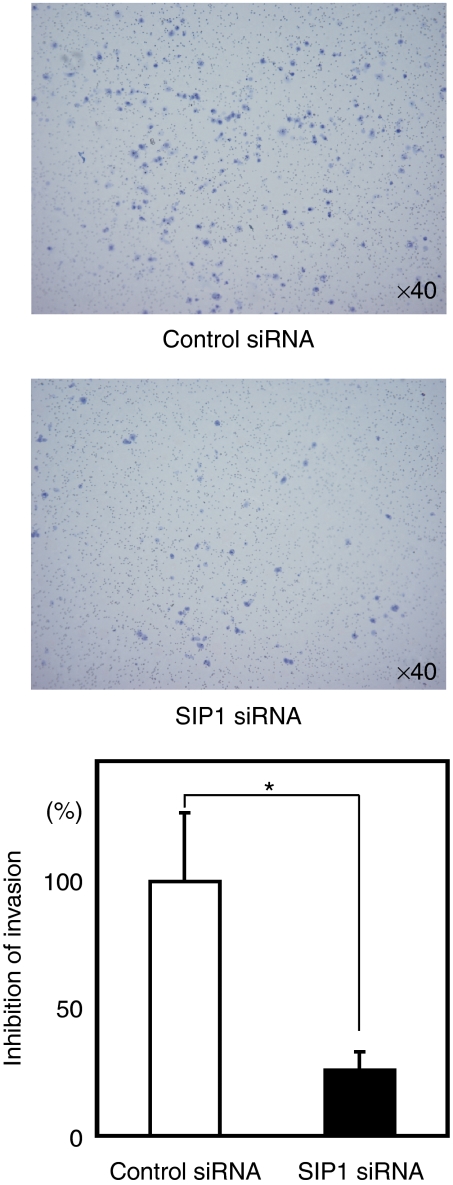
The EMT regulator gene SIP1 involves gastric cancer cell invasion. Matrigel invasion assays indicate that *SIP1* siRNA transfection inhibits migration of HSC60 cells. Results were calculated as mean+s.d. values from triplicate measurements of three separate experiments. ^*^P<0.05.

Finally, to investigate the biological implications of the Hh–EMT pathway in diffuse-type GC, Matrigel invasion assays were performed. Although no cell growth inhibition was observed at 24 h after *SIP1* siRNA transfection, the number of invaded HSC60 cells was significantly decreased after *SIP1* siRNA transfection compared with a control siRNA (Figure 7), suggesting that SIP1 was able to regulate genes critical to the invasion of diffuse-type GC cells.

## Discussion

**Figure 8:**
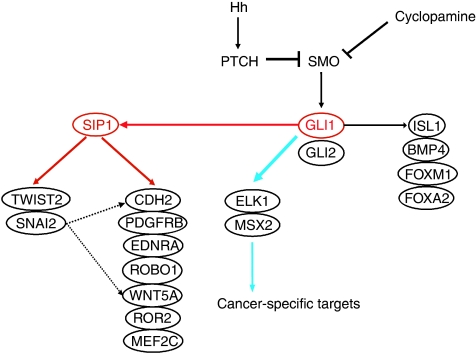
Hypothetical schema of cross talk between Hh and EMT signal pathways in both pit cells and diffuse-type gastric cancers. A transcriptional cross talk between Hh and EMT is indicated by red arrows, and that between Hh and cancer-specific genes (*ELK1* and *MSX2*) is indicated by blue arrows. Broken arrows that are headed from SNAI2 to CDH2 and WNT5A are based on a previous report (Taki *et al*, 2003).

Our present results suggest the presence of cross talk between Hh and EMT in gastric pit cells and in diffuse-type GC (Figure 8). The reason why gastric pit cells exhibit the mesenchymal phenotype, despite differentiating or differentiated epithelial cells, is unknown. Recently, we reported that GSDM/GSDMA was a target of RUNX3 and LMO1 in transforming growth factor-beta (TGF-*β*) signalling for apoptosis of gastric pit cells (Saeki *et al*, 2007). Transforming growth factor-beta is well known for inducing EMT as well as apoptosis of epithelial cells (Zavadil and Böttinger, 2005). Therefore, EMT may have a role in migration of the differentiating pit cells toward the gastric lumen.

To address the molecular mechanism of the cross talk between Hh and EMT, chromatin immunoprecipitation (ChIP)-on-chip analysis is a hopeful tool for showing an *in vivo* direct interaction of the *SIP* promoter with GLI1, or of SIP1-downstream genes with SIP1. Although a GLI1 binding consensus sequence can be found about 1 kb upstream on the *SIP1* promoter, there is no report showing a GLI1 antibody suitable for ChIP. As shown in Figure 6B, mRNAs of two GC-linked DNA hypomethylated oncogene candidates, *ELK1* and *MSX2*, were clearly reduced by cyclopamine treatment and by *GLI1* siRNA transfection. These results suggest that GLI1 or other GLI1-target transcriptional factors, including ISL1, FOXM1, FOXA2, and SIP1, may regulate both *ELK1* and *MSX2*. These issues remain, however, for future studies.

More than 70% of advanced GC patients show poor prognosis (Wohrer *et al*, 2004; Lordick and Siewert, 2005). Therefore, a new strategy or drug development is eagerly awaited. Advanced diffuse-type GC is known to frequently show peritoneal metastasis within 3 years. Our previous study (Fukaya *et al*, 2006) and this study show that the Hh signal is activated in most diffuse-type GCs, and that the Hh signal-specific inhibitor, cyclopamine, effectively suppressed invasion as well as growth of Hh signal-activated GC cells (Figure 7 and Fukaya *et al*, 2006). Therefore, diffuse-type GC could be a therapeutic target of Hh-pathway-specific inhibitors, especially for the protection of peritoneal recurrence. Our proposed extensive Hh signal pathway containing an EMT pathway could contribute to new drug development. As shown in Figure 8, two transcription factors, GLI1 and SIP1, seem to be the key molecules for the Hh–EMT pathway in diffuse-type GC, because GLI1 regulates cell growth-related genes, such as *ELK1*, *MSX2*, and *FOXM1* (Kim *et al*, 2006; Yoshida *et al*, 2007), and because SIP1 regulates cell invasion-related genes such as EMT regulators (*TWIST2* and *SNAI2*) (Taki *et al*, 2003; Yang *et al*, 2004), *PDGFRB* (Singh *et al*, 2007), and *WNT5A* (Kurayoshi *et al*, 2006). An increasing number of genes including oncogenes are found to be normally methylated at promoters but hypomethylated and activated in the corresponding tumours (Feinberg *et al*, 2006). These include *R-RAS* and *MASP* in GC (Akiyama *et al*, 2003; Nishigaki *et al*, 2005), *MAGE1* in melanoma (De Smet *et al*, 1996), *S100A4* in colon cancer (Nakamura and Takenaga, 1998), *PAX2* in endometrial cancer (Wu *et al*, 2005), *DNMT3A* in testicular germ cell tumours (Ishii *et al*, 2007), and various genes in pancreatic cancer (Sato *et al*, 2003). *ELK1* and *MSX2*, encoding transcription factors, may belong to this class of oncogene. Double transfection of *ELK1* and *MSX2* siRNAs to diffuse-type GC cells showed strong cell growth inhibition compared with a single transfection (Figure 6C and D, and Supplementary Figure 4), suggesting that ELK1 and MSX2 act independently on GC cell growth. Therefore, a genome-wide search of the transcriptional downstream target gene of ELK1 and MSX2 in addition to GLI1 and SIP1 is thought to be important for future identification of molecular targets of diffuse-type GC.

In summary, our study showed that genome-wide mRNA expression profiling provides some hints for identifying specific signal pathways, their cross talk, and some molecules aberrantly expressed in the pathways in a certain type of cancer. However, the sensitivity varies among microarray platforms, and most or all major platforms have an insufficient sensitivity to detect a key molecule, such as a growth factor or transcription factor, which acts with a small expression. Improvement of the sensitivity is needed for future cancer transcriptome.
