# Supplementary figures and images for: Cross talk between hedgehog and epithelial–mesenchymal transition pathways in gastric pit cells and in diffuse-type gastric cancers
Source: Br J Cancer. 2008 Dec 23;100(2):389–98. doi: 10.1038/sj.bjc.6604846 (PMC2634717; doi:10.1038/sj.bjc.6604846)

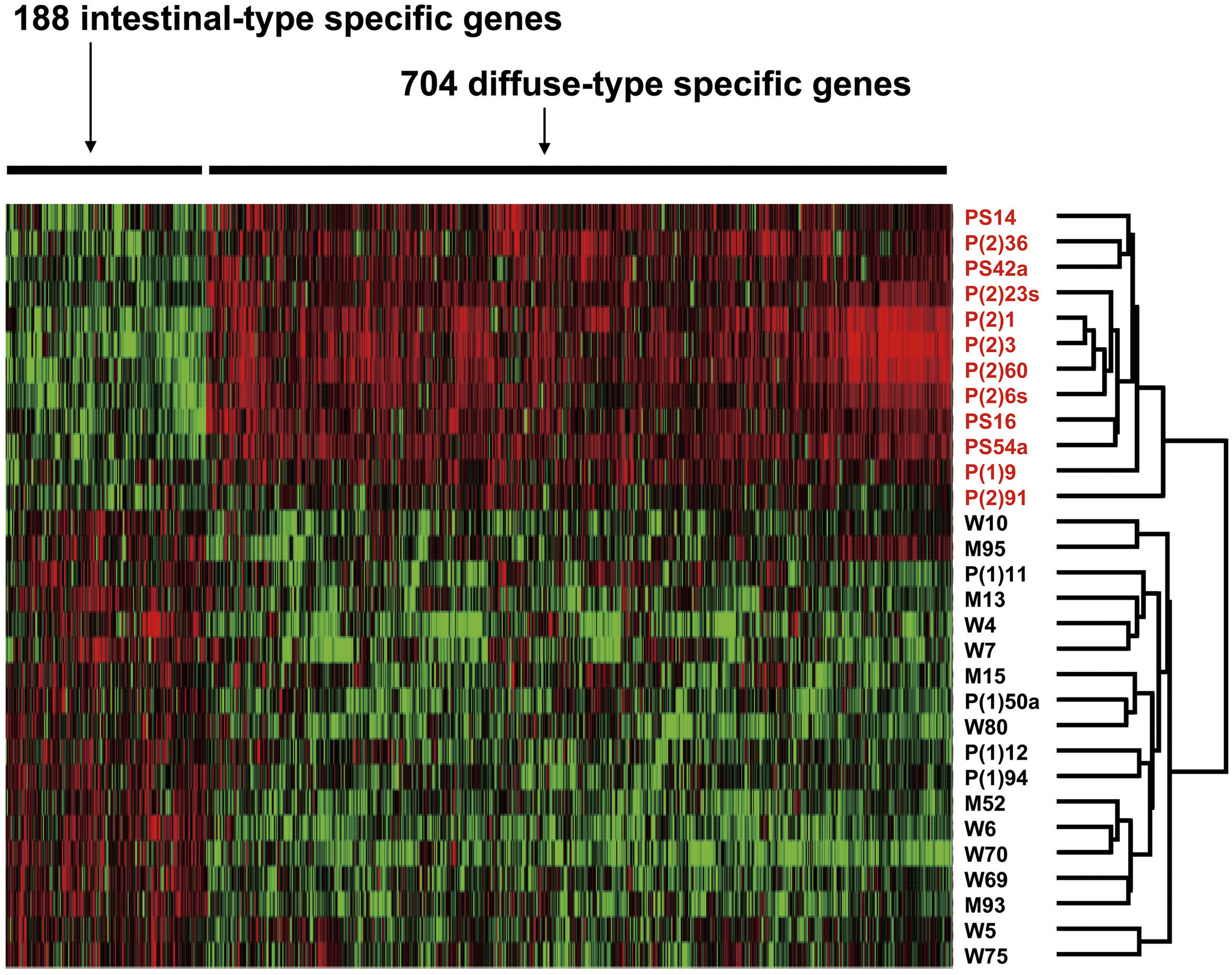

Supplement: Supplementary Figure 1 [file 6604846x1.tif]

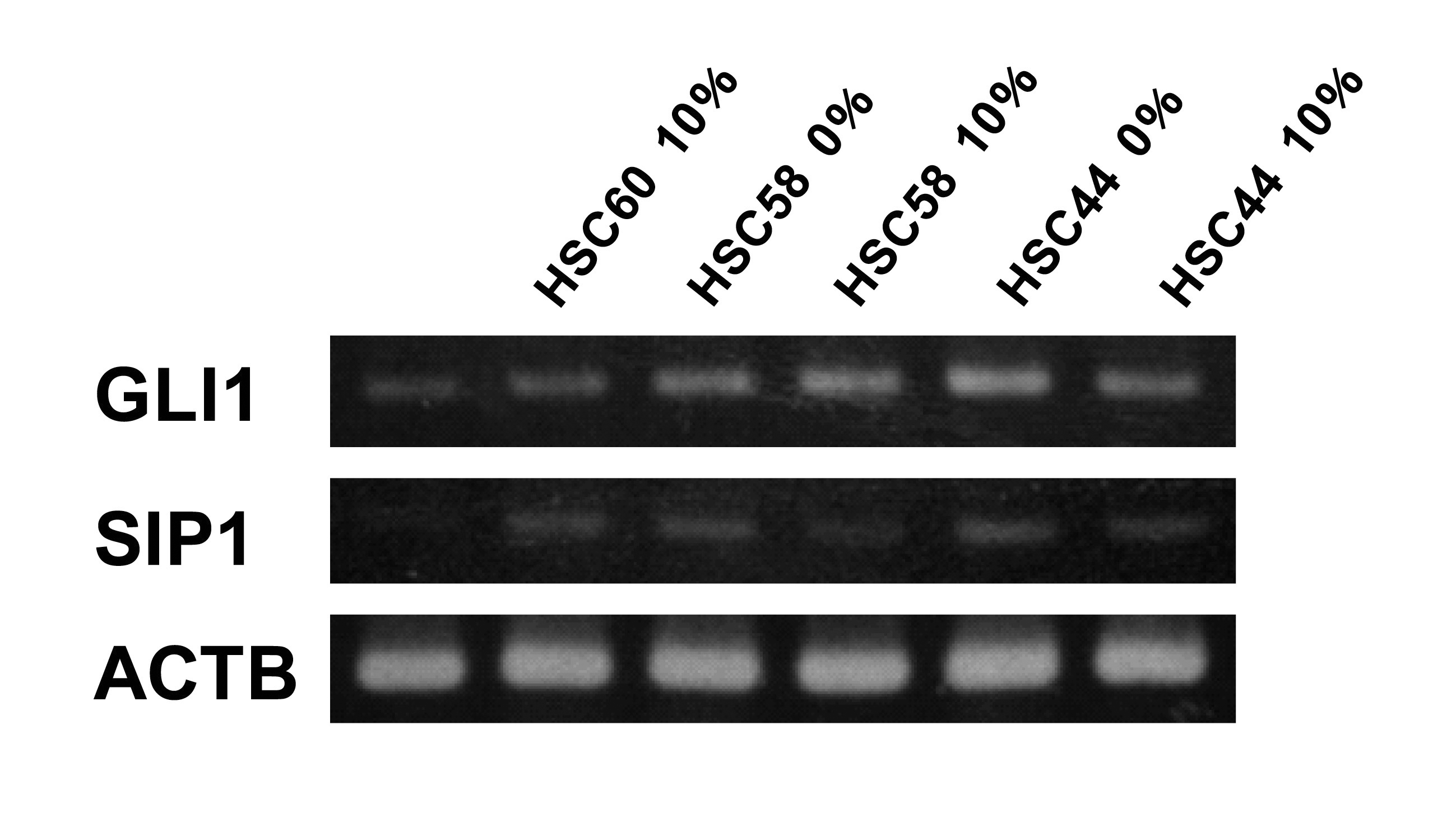

Supplement: Supplementary Figure 2 [file 6604846x2.tif]

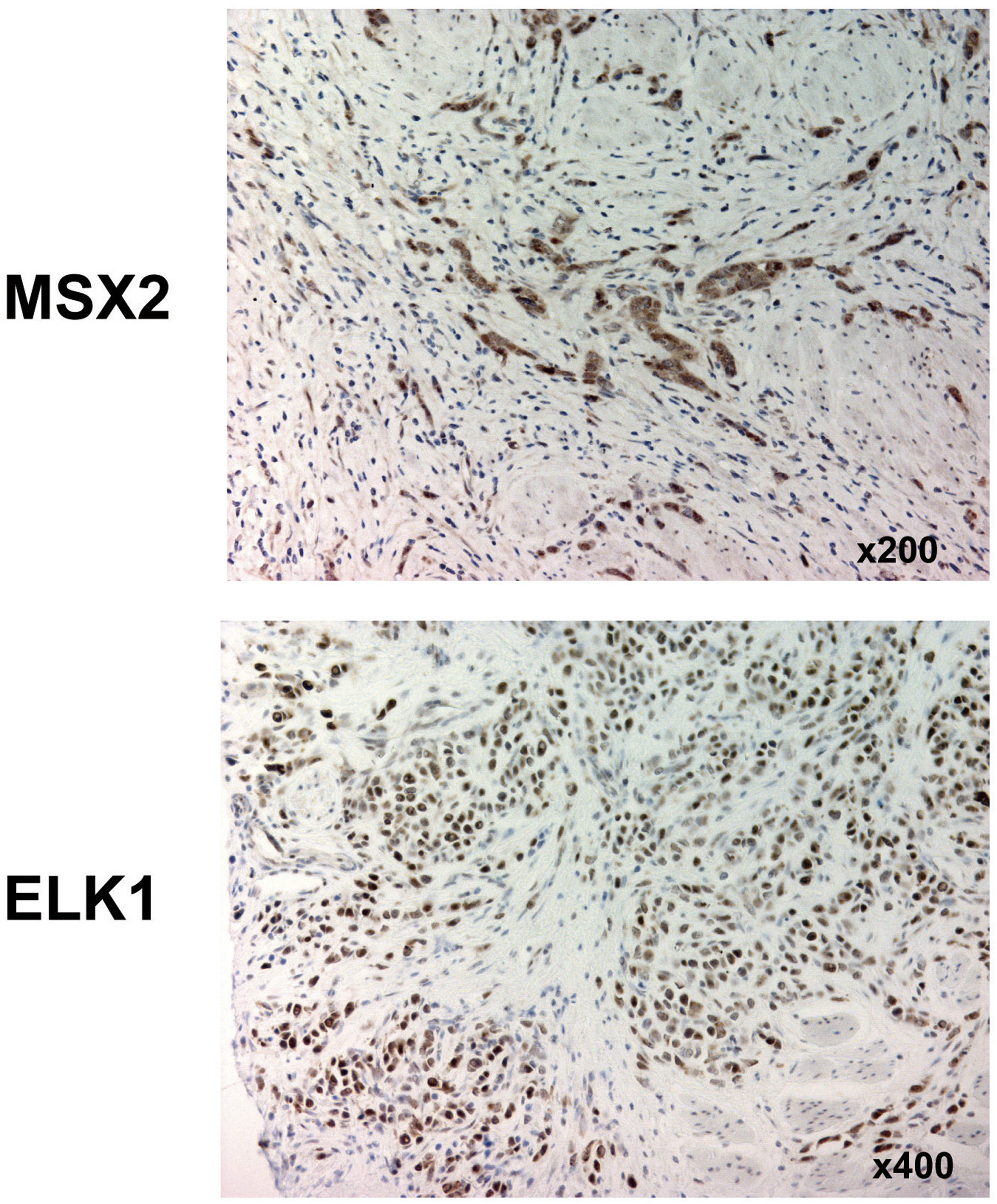

Supplement: Supplementary Figure 3 [file 6604846x3.tif]

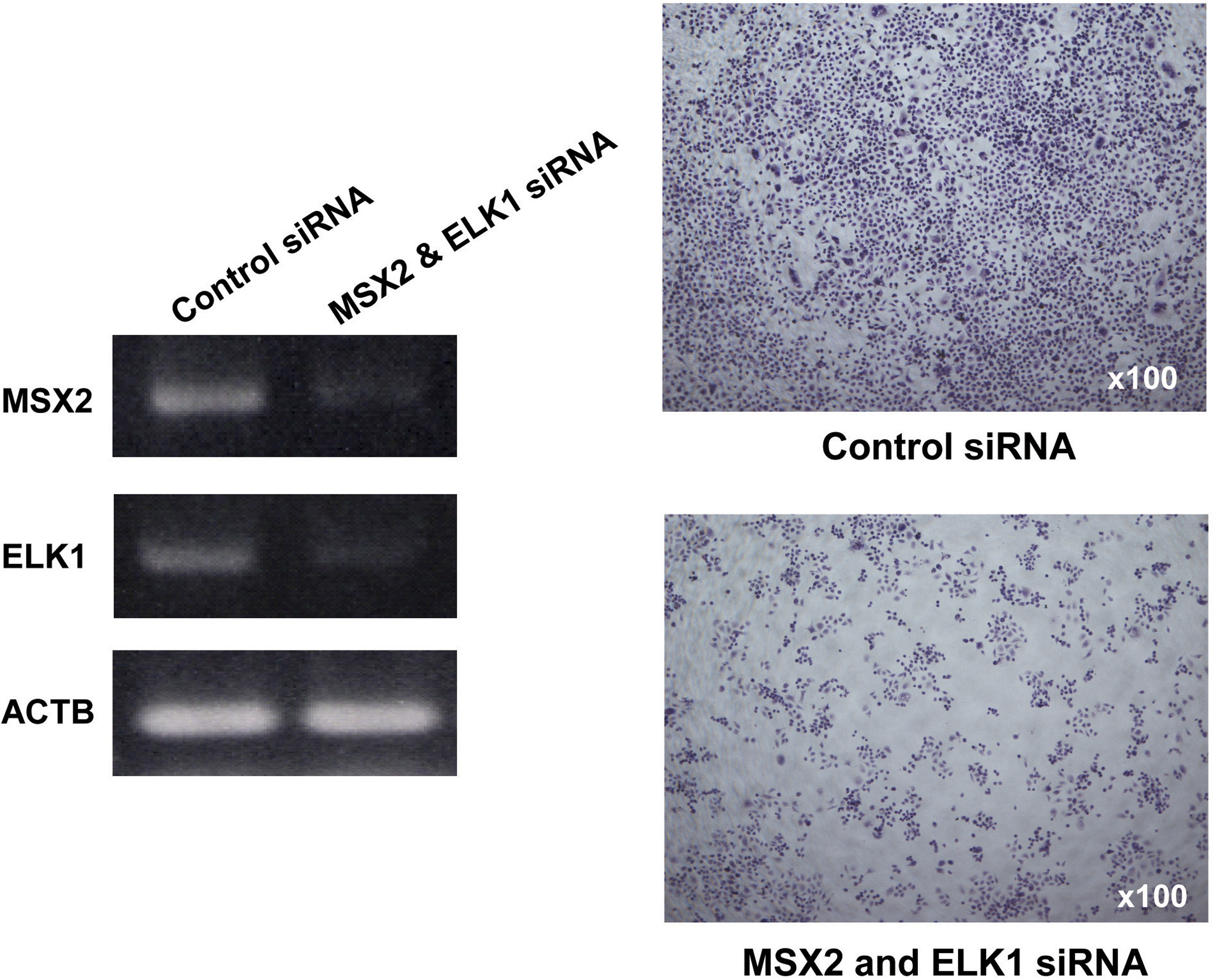

Supplement: Supplementary Figure 4 [file 6604846x4.tif]
