# Supplementary material for: Cross talk between hedgehog and epithelial–mesenchymal transition pathways in gastric pit cells and in diffuse-type gastric cancers
Source: Br J Cancer. 2008 Dec 23;100(2):389–98. doi: 10.1038/sj.bjc.6604846 (PMC2634717; doi:10.1038/sj.bjc.6604846)
Supplement: Supplementary Table [file 6604846x5.doc]

Supplemental Table. Primer sequences

|  |  |  |  |
| --- | --- | --- | --- |
| Human |  |  |  |
|  | Forward primer |  |  |
|  | Reverse primer |  |  |
|  |  |  |  |
| Gene | RT-PCR |  | Real-time　RT-PCR |
| SHH | 5'-ACCATTCTCATCAACCGGGT-3' |  |  |
|  | 5'-ATTTGGTAGAGCAGCTGCGA-3' |  |  |
|  |  |  |  |
| IHH | 5'-GAGACTCTTTCACAGCTTGG-3' |  |  |
|  | 5'-GCTTGCAGCTCTATGACTAC-3' |  |  |
|  |  |  |  |
| DHH | 5'-TGGCATGCATTGGTACTCTC-3' |  |  |
|  | 5'-TATCACCTCCTCTCAGTACG-3 |  |  |
|  |  |  |  |
| BOC | 5'-TAACAGGAGTCACCCAGGAA-3' |  |  |
|  | 5'-GCTGTACACATCTTGGTCTG-3' |  |  |
|  |  |  |  |
| PTCH | 5'-CAAATCCACACCAGCACCTT-3' |  |  |
|  | 5'-GTCTGAGGTCACTATGCTGT-3' |  |  |
|  |  |  |  |
| SMO | 5'-CTGCACACACTCACCTCTAA-3' |  |  |
|  | 5'-AAGCTTTCTTGCCTGGCTGA-3' |  |  |
|  |  |  |  |
| GLI1 | 5'-TCTCAAAGTGGGAGGCACAA-3' |  | 5'-TCTCAAAGTGGGAGGCACAA-3' |
|  | 5'-GGTAGGGATCTCAGTAAGAC-3' |  | 5'-CCCTTAGGAAATGCGATCTG-3' |
|  |  |  |  |
| GLI2 | 5'-TTATGGGCATCCTCTCTGGT-3' |  | 5'-TTATGGGCATCCTCTCTGGT-3' |
|  | 5'-CGGAGCAGAGTATCCAGTAT-3' |  | 5'-CGGAGCAGAGTATCCAGTAT-3' |
|  |  |  |  |
| ISL1 | 5'-CCTCTATTTTGCCACAAGCG-3' |  |  |
|  | 5'-GTGGCAAGTCTTCCGACAAT-3' |  |  |
|  |  |  |  |
| BMP4 | 5'-CACCTCATCACACGACTACT-3' |  |  |
|  | 5'-TCCAGTATCCCCAAAGCCTGT-3' |  |  |
|  |  |  |  |
| FOXM1 | 5'-GTCTCTTACCTTCCCTGATC-3' |  |  |
|  | 5'-AAGCTGACTTGGAAACACGG-3' |  |  |
|  |  |  |  |
| FOXA2 | 5'-CTTCAAGCACCTGCAGATTC-3' |  |  |
|  | 5'-AGACCTGGATTTCACCGTGT-3' |  |  |
|  |  |  |  |
| SIP1 | 5'-TGCTCGCACTACAATGCATC-3' |  | 5'-CATCCTCACACCTCTGCATT-3' |
|  | 5'-ACAGGGTGAGCTTAACACTG-3' |  | 5'-GGCCAGTTTGATCCCAGTAT-3' |
|  |  |  |  |
| SNAI2 | 5'-TAGGAAGAGATCTGCCAGAC-3' |  | 5'-TAGGAAGAGATCTGCCAGAC-3' |
|  | 5'-CCCCAAGGCACATACTGTTA-3' |  | 5'-CCCCAAGGCACATACTGTTA-3' |
|  |  |  |  |
| TWIST1 | 5'-CATCCTCACACCTCTGCATT-3' |  |  |
|  | 5'-GGCCAGTTTGATCCCAGTAT-3' |  |  |
|  |  |  |  |
| TWIST2 | 5'-CTTATGTTTGGGGGGAGGTT-3' |  | 5'-CTTATGTTTGGGGGGAGGTT-3' |
|  | 5'-TAGCCAAGCAATCACGGAGA-3' |  | 5'-TAGCCAAGCAATCACGGAGA-3' |
|  |  |  |  |
| CDH2 | 5'-GGCATAGTCTATGGAGAAGT-3' |  |  |
|  | 5'-GCTGTTGTCAGAAGTCTCTC-3' |  |  |
|  |  |  |  |
| VIM | 5'-ATTGCCACCTACAGGAAGCT-3' |  |  |
|  | 5'-GCAGAAAGGCACTTGAAAGC-3' |  |  |
|  |  |  |  |
| FN1 | 5'-AGCAGACCCAGCTTAGAGTT-3' |  |  |
|  | 5'-GCAGAAGTGTTTGGGTGACT-3' |  |  |
|  |  |  |  |
| SMTN | 5'-TTGAGAAGCTGGAGAAGGAG-3' |  |  |
|  | 5'-TAAGCTGCCCATAGTCGAAG-3' |  |  |
|  |  |  |  |
| LMOD | 5'-AGAGGAGACAGACACACACT-3' |  |  |
|  | 5'-GTATCCACCTCACAGAGTTG-3' |  |  |
|  |  |  |  |
| MEF2C | 5'-TCACTGTTGTGCTCCTTTGC-3' |  |  |
|  | 5'-ATGAGTGCCATACGCCAATG-3' |  |  |
|  |  |  |  |
| PDGFRB | 5'-ACTGCCCAGACCTAGCAGTG-3' |  |  |
|  | 5'-CAGGGAAGTAAGGTGCCAAC-3' |  |  |
|  |  |  |  |
| EDNRA | 5'-TGAAATTGCCAGGTTGTCTG-3' |  |  |
|  | 5'-GATGCGCCAGTGGAATAATAG-3' |  |  |
|  |  |  |  |
| SLIT2 | 5'-CCTACTGTGAATGCAGCAGT-3' |  |  |
|  | 5'-CCGTCAGTGCATTCGAAAGA-3' |  |  |
|  |  |  |  |
| ROBO1 | 5'-GTTGTGGCCAATGTCGAAAC-3' |  |  |
|  | 5'-GTTTTGGTGAACACCAGCCT-3' |  |  |
|  |  |  |  |
| WNT5A | 5'-CCGGTACTAGCTAACTCCAA-3' |  |  |
|  | 5'-CACCATTCCACAGAGAGAGA-3' |  |  |
|  |  |  |  |
| ROR2 | 5'-GGGTTTGGAATGTTCAGAGG-3' |  |  |
|  | 5'-CTGTGGGTCTGTGTGTAACA-3' |  |  |
|  |  |  |  |
| ELK1 | 5'-TCTCCTTCCAGTTTCCATCC-3' |  | 5'-TCTCCTTCCAGTTTCCATCC-3' |
|  | 5'-ATAAACCGCCCCTACCATTG-3' |  | 5'-ATAAACCGCCCCTACCATTG-3' |
|  |  |  |  |
| MSX2 | 5'-CCTGTTGAGAGGAATTGATGG-3' |  | 5'-CCTGTTGAGAGGAATTGATGG-3' |
|  | 5'-AAAGGTATACCGGAGGGAGG-3' |  | 5'-AAAGGTATACCGGAGGGAGG-3' |
|  |  |  |  |
| ACTB | 5'-TCATCACCATTGGCAATGAG-3' |  |  |
|  | 5'-CACTGTGTTGGCGTACAGGT-3' |  |  |
|  |  |  |  |
| GAPDH |  |  | 5'-CGACCACTTTGTCAAGCTCA-3' |
|  |  |  | 5'-TCTACATGGCAACTGTGAGGA-3' |
|  |  |  |  |
|  |  |  |  |
| Mouse |  |  |  |
|  | Forward primer |  |  |
|  | Reverse primer |  |  |
|  |  |  |  |
| Gene | RT-PCR |  | Real-time　RT-PCR |
| Gli1 | 5'-GCCATGAAACTTTCACCGTG-3' |  | 5'-TCTGGGGGGTTACCAAGTTA-3' |
|  | 5'-CTCATGTTACCCACTGCCAT-3' |  | 5'-CACAAAGTCCAGCTGAGTGT-3' |
|  |  |  |  |
| Gli2 | 5'-AGGTATACAGTGAAGCCCTC-3' |  | 5'-TGCTGTGGACTAGGAATAGG-3' |
|  | 5'-ACCATGCATGTCATCACTGG-3' |  | 5'-AACCTTCCGCTCAACCACAA-3' |
|  |  |  |  |
| Sip1 | 5'-CGCACTACAGTGCATCAGTA-3' |  | 5'-CGCACTACAGTGCATCAGTA-3' |
|  | 5'-GCTCACCGTGTTCTTTGAAG-3' |  | 5'-GCTCACCGTGTTCTTTGAAG-3' |
|  |  |  |  |
| Snai2 | 5'-TTGGAAGAGGAGGGAAAGAC-3' |  | 5'-TTGGAAGAGGAGGGAAAGAC-3' |
|  | 5'-CACTTGGAGGGGCATTGTAT-3' |  | 5'-CACTTGGAGGGGCATTGTAT-3' |
|  |  |  |  |
| Twist2 | 5'-CTCCTGGGTTTTAGTTTGGG-3' |  | 5'-CTCCTGGGTTTTAGTTTGGG-3' |
|  | 5'-GAATTCCAGGCTTCCTCGAA-3' |  | 5'-GAATTCCAGGCTTCCTCGAA-3' |
|  |  |  |  |
| Cdh2 | 5'-GTGATACGCTTCAACCCACT-3' |  |  |
|  | 5'-CCATTTTTACAGCGAGGTGG-3' |  |  |
|  |  |  |  |
| Pdgfrb | 5'-GACTTGGAGTGACAGTGAGT-3' |  |  |
|  | 5'-CTTCCTCTCATTGCCCATCT-3' |  |  |
|  |  |  |  |
| Ednra | 5'-AGTATGCCTGAGACTTCCAG-3' |  |  |
|  | 5'-AGGTTCATGGCAAGTGTAAC-3' |  |  |
|  |  |  |  |
| Robo1 | 5'-GCTCTGTGACTGTAGGTTTC-3' |  |  |
|  | 5'-TGGGGCTTCTTCAAATTAGC-3' |  |  |
|  |  |  |  |
| Wnt5a | 5'-ATTTCCCCTCAGCTACAATG-3' |  |  |
|  | 5'-TGTTAAACTCCACTCTGCTC-3' |  |  |
|  |  |  |  |
| Ror2 | 5'-TGAACACTTGGGAGTGCCTT-3' |  |  |
|  | 5'-CTGCTTTCTGTACAACGTTG-3' |  |  |
|  |  |  |  |
| Mef2c | 5'-CACTGTTGTGACCCTTTGCA-3' |  |  |
|  | 5'-ATGAGTGCCATACGCCAATG-3' |  |  |
|  |  |  |  |
| Smtn | 5'-AAAGTCCTAACCCCTGCTTG-3' |  |  |
|  | 5'-CAAAACGCTGCGTGTGTACA-3' |  |  |
|  |  |  |  |
| Actb | 5'-CTACAAATGTGGCTGAGGC-3' |  |  |
|  | 5'-ACAGAAGCAATGCTGTCAC-3' |  |  |
|  |  |  |  |
| Gapdh |  |  | 5'-CTGCGACTTCAACAGCAACT-3' |
|  |  |  | 5'-TCTTGCTCAGTGTCCTTGCT-3' |
|  |  |  |  |
