# Supplementary material for: Cross talk between hedgehog and epithelial–mesenchymal transition pathways in gastric pit cells and in diffuse-type gastric cancers
Source: Br J Cancer. 2008 Dec 23;100(2):389–98. doi: 10.1038/sj.bjc.6604846 (PMC2634717; doi:10.1038/sj.bjc.6604846)
Supplement: Supplementary Figure Legends [file 6604846x6.doc]

**Legends for Supplemental Figures**

**Supplemental Figure 1** Supervised clustering analysis of 892 specifically expressed genes in 18 intestinal-type or 12 diffuse-type gastric cancers. By Wilcoxon u-test (p<0.05) from genes with more than 2-fold change in average, 188 genes were selected as specific genes for 18 intestinal-type gastric cancers, and 704 genes were selected as specific genes for 12 diffuse-type gastric cancers. A result of a two-dimensional hierarchical clustering analysis of the 892 selected genes is shown.

**Supplemental Figure 2** Expression of *GLI1* and *SIP1* in gastric cancer cell lines after serum depletion. RT-PCR analyses of *GLI1* and *SIP1* in three gastric cancer cell lines (HSC60, HSC58, and HSC44) at 24 hours after serum depletion were performed. Expression of both the two genes is never increased in a serum free culture.

**Supplemental Figure 3** Immunohistochemistry of ELK1 and MSX2 in diffuse-type gastric cancer tissues. Both ELK1 and MSX2 are localized preferentially in the nuclei of diffuse-type gastric cancer cells.

**Supplemental Figure 4** Cell growth inhibition by double transfection of *ELK1* siRNA and *MSX2* siRNA in gastric cancer cells. Double transfection of *ELK1* siRNA and *MSX2* siRNA strongly inhibits cell growth of HSC60 cells compared with a single transfection (Fig. 6C).
